# Effect of gallic acid on chronic restraint stress-induced anxiety and memory loss in male BALB/c mice

**DOI:** 10.22038/ijbms.2018.31230.7523

**Published:** 2018-12

**Authors:** Azadeh Salehi, Zahra Rabiei, Mahbubeh Setorki

**Affiliations:** 1 Department of Biology, Izeh Branch, Islamic Azad University, Izeh, Iran; 2 Medical Plants Research Center, Basic Health Sciences Institute, Shahrekord University of Medical Sciences, Shahrekord, Iran

**Keywords:** Anxiety, Gallic acid, Malondialdehyde, Oxidative stress, Passive avoidance memory

## Abstract

**Objective(s):**

Long-term exposure to stress leads to memory deficits and certain mood disorders such as depression and anxiety. We aimed to study the effect of gallic acid (GA) on chronic restraint stress (CRS) induced anxiety and memory deficits in male BALB/c mice.

**Materials and Methods:**

Ninety male BALB/c mice were assigned to nine groups including caged control (CC): food-water deprived (FWD), under chronic restraint stress (CRS), CRS+ gallic acid (5, 10, and 20 mg/kg), and gallic acid (5, 10, and 20 mg/kg). Behavioral assays were performed after 21 days of daily treatment with CRS and GA. Serum and brain levels of malondialdehyde (MDA) and total antioxidant capacity (TCA) and serum corticosterone level were also measured.

**Results:**

Treatment of CRS mice with GA significantly improved passive avoidance memory in the shuttle box and ameliorated anxiety-like behaviors in the elevated plus maze (EPM) and open filed test (OFT). GA treatment significantly reduced elevated levels of serum and brain MDA and increased brain TCA. CRS and GA did not affect serum corticosterone levels. Treatment of healthy mice with GA had some adverse effects and induced some anxiety and oxidative stress.

**Conclusion:**

GA exerted protective effects against stress-induced mood and memory deficit disorders.

## Introduction

Unpredictable and uncontrollable stressful events affect the functions of all organs and systems in the body by influencing the physiological processes of the central nervous system (CNS) (1, 2). The hypothalamic-pituitary-adrenal (HPA) axis and sympathetic nervous system (SNS) serve as the major components of the CNS in response to stress. When a stressful situation happens, the hypothalamus releases corticotropin-releasing hormone (CRH), and *arginine vasopressin* (AVP), both of these neurohormones stimulate the secretion of adrenocorticotropic hormone (ACTH) from the posterior pituitary and activate the noradrenergic neurons of the locus coeruleus-noradrenergic system (LC-NA system*)*. LC-NA system mediates alert and escapes behaviors in response to epinephrine and norepinephrine, while ACTH stimulates the adrenal glands to produce glucocorticoids (3).

Cortisol (hydrocortisone) is the major glucocorticoid in humans and most other mammals, while corticosterone is the major circulating glucocorticoid in birds, reptiles, amphibians, and rodents such as rat and mouse. Small elevation of blood cortisol is associated with some positive effects such as a rapid increase of body’s energy to cope with the stressful situation, increase of memory performance, boosting of immune system function, the decrease of pain sensitivity, and maintenance of body’s homeostatic balance (4).

Although cortisol represents an important part of the body’s response to stress, it is important to *restore* the body to a state of relaxation so that it can normalize shortly after stress. Cortisol prevents prolonged exposure of tissues to high catabolic and immunosuppressive activities through negative feedback (5). Unfortunately, in the current stressful environment, the body’s response to stress is activated too sustained and too frequently and there is often no time to return to normal state, and therefore stress is very likely to turn into chronic stress (6). It has been shown that high long-term circulatory cortisol levels (induced by certain factors such as chronic stress) have adverse effects such as physical and mental fatigue, cognitive dysfunction, thyroid disorders, blood sugar imbalance, loss of bone-muscle mass, hypertension, and weakness of immune system (7, 8).

Studies on animal models have indicated that exposure to chronic stress is associated with mood disorders, memory deficit, and *c*ognitive decline (1, 6). Interestingly, memory processes and their formation are differently influenced by chronic stress, for instance, working memory is influenced by acute and mild stress (9) and hypothalamus-dependent declarative memory is affected by chronic stress (1). 

Given the role of chronic stress in the development of mood disorders such as depression and anxiety and its adverse effects on cognitive functions, seeking out certain strategies to decrease these effects has attracted attention. Medicinal plants can prevent the development of chronic stress-induced cognitive and mood disorders through decreasing oxidative stress parameters and serum levels of glucocorticoids (10). 

Gallic acid (GA), also known as 3, 4, 5-trihydroxybenzoic acid, is a potential antioxidant from the family of phenolic compounds that is found in wheat, hazelnuts, tea leaves, oak wood skin, and some other plants (11). Pharmacological studies have demonstrated the neuroprotective effects of GA against 6-hydroxydopamine-induced Parkinsonism (12), beta-amyloid-induced neurotoxicity and brain inflammation (13), streptozocin-induced oxidative stress and memory loss (14), and transient cerebral ischemia (15). The aim of our study was to investigate the effects of GA on anxiety and memory deficits and anxiety induced by chronic stress in male mice.

## Materials and Methods

***Evaluation of in vitro antioxidant activity of gallic acid (GA)***

*DPPH radical scavenging activity*

Briefly, 1 ml of 0.1 mM DPPH solution (prepared in 95% ethanol) was added to 1 ml of GA at different concentrations (1-10 µg/ml) and incubated for 15 min in the dark at room temperature, and then the absorbance was read at 517 nm against a blank sample. The blank sample was prepared using distilled water instead of GA. DPPH radical scavenging activity was calculated using the following formula. DPPH radical scavenging activity (%) = [(A_blank_-A_sample_) /A_blank_] × 100. The IC_50_ value was obtained by plotting a graph of concentration (X-axis) against the percentage of inhibition (Y-axis) (16).

*Metal chelating activity*

Briefly, 1 ml of GA at various concentrations (100-10,000 µg/ml) was added to 3.7 ml of distilled water, and then mixed with 0.1 ml of 2 mM FeCl_2_ and 0.2 ml of 5 mM ferrozine, and after approximately 20 min, the absorbance was measured at 562 nm against a blank sample. The blank sample was prepared using distilled water instead of GA. Metal chelating activity was calculated using the following formula. Metal chelating activity (%) = [(A_blank_-A_sample_) /A_blank_] × 100. The IC_50_ value was calculated from the plot of the chelating activity against GA concentrations (16).

*Ferric reducing activity*

Briefly, 2 ml of gallic acid (100-1000 µg/ml) was mixed with 2 ml of phosphate buffer (0.2 M, pH 6.6) and 2 ml of 1% potassium ferricyanide. After incubation at 50 ^°^C for 20 min, 2 ml of 10% Trichloroacetic acid (TCA) was added to the mixture. Following centrifugation at 3000 rpm for 10 min, 2 ml of the supernatant was mixed with 2 ml of distilled water and 0.5 ml of 0.1% FeCl_3_. Then the optical absorbance was recorded at 700 nm (16).

*ABTS radical scavenging activity*

Working solution of ABTS was prepared by reacting 10 ml of 7.4 mM ABTS with 10 ml of 2.6 mM potassium persulfate for 12 hr in the dark at room temperature. After the incubation period, the freshly prepared ABTS solution was diluted with methanol to obtain an absorbance of 1.1±0.02 at 734 nm. Then, 2850 μl of ABTS solution was mixed with 150 μl of GA solution at different concentrations (1-10 µg/ml), and after incubation at 23±2 ^°^C for 1 hr, the absorbance was recorded. The blank sample was prepared using 150 μl of distilled water instead of GA. ABTS scavenging activity was determined using the following formula. ABTS scavenging activity (%) = [(A_blank_-A_sample_) /A_blank_] × 100. The IC_50_ value was determined from the plot of the scavenging activity against GA concentrations (16).

*Hydroxyl radical scavenging activity*

First, one ml of 1.865 mM 1, 10-phenanthroline solution was added to 2 ml of the GA solution (100-1000 µg/ml) and mixed. Then, 1 ml of H_2_O_2_ (3% v/v) was added to the mixture and after 60 min of incubation at 37 ^°^C in a water bath, the absorbance was recorded at 536 nm. The solution containing GA without H_2_O_2_ was considered as blank and solution containing H_2_O_2 _without GA was considered negative control. Hydroxyl radical scavenging activity was determined using the following formula. Hydroxyl radical scavenging activity (%) = [(A_sample_-A_negative-control_)/ (A_blank_-A_negative_-_control_)] ×100. The IC_50_ value was determined from the plot of the scavenging activity against the sample concentration (16).

***Animals and grouping***

Ninety male mice weighing 25–30 g were housed under 23±2 ^°^C and 12 hr light/12 hr dark and fed the same food and water. Mice were then assigned to nine groups of 10 each Randomly: Group 1 (caged control (CC)), only *received *IP *injection of* normal saline for 21 days; Group 2 (food-water deprived (FWD)), deprived of food and water for 6 hr daily and received IP injection of normal saline for 21 days; Group 3 (under chronic restraint stress (CRS)), underwent 6 hr daily restraint stress and received IP injection of normal saline for 21 consecutive days; Groups 4-6 (intervention group), underwent CRS and received IP injection of gallic acid at doses of 5, 10, and 20 mg/kg, respectively, 30 min before CRS for 21 days; and Groups 7-9 (GA treated group), *received *GA at doses of 5, 10, and 20 mg/kg, respectively, for 21 consecutive days. After 21 days of treatment, passive avoidance memory and anxiety were measured by the elevated plus maze, open-field, and shuttle box tests. After behavioral examination, blood samples were taken from the animal hearts under deep anesthesia and centrifuged at 1500 rpm for 10-15 min. To separate the sera, isolated sera were then stored at -80 ^°^C until biochemical measurements. Brain tissue was also removed and stored at -80 ^°^C until biochemical analysis. All animal procedures were based on the Guideline for the Care and Use of Laboratory Animals. The study was reviewed and approved by the Ethics Committee of the Islamic Azad University of Izeh. 

*The elevated plus maze (EPM) test*

An apparatus called the elevated plus maze was used to measure anxiety. This apparatus has two opposite open arms, two opposite closed arms, and a central sheath elevated 50 cm above the floor. This test was performed in a relatively dark, silent chamber, and each mouse was placed gently in the center of the device facing the open arm and allowed to explore for 5 min. The number of entries and time spent in each arm were recorded (17).

**Figure 1:**
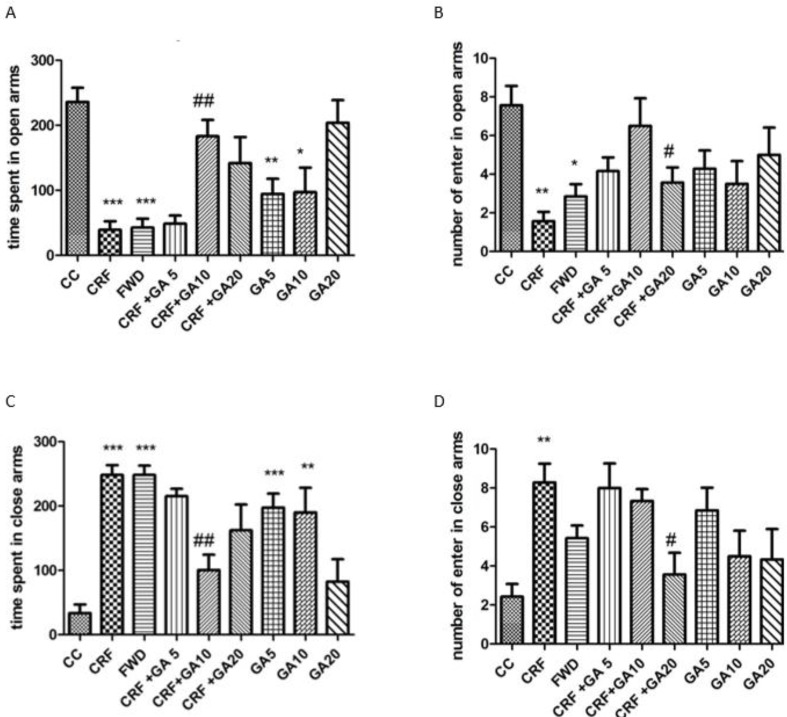
Number of entries in open arms (a), percentage of time spent in open arms (b), number of entries in closed arms (c), and percentage of time spent in closed arms (d) of the Elevated Plus Maze; * Shows significant difference compared to control (CC) and # shows significant difference compared to the chronic restraint stress (CRS) group (* *P*<0.05, ** *P*<0.01, and *** *P*<0.001)

**Figure 2:**
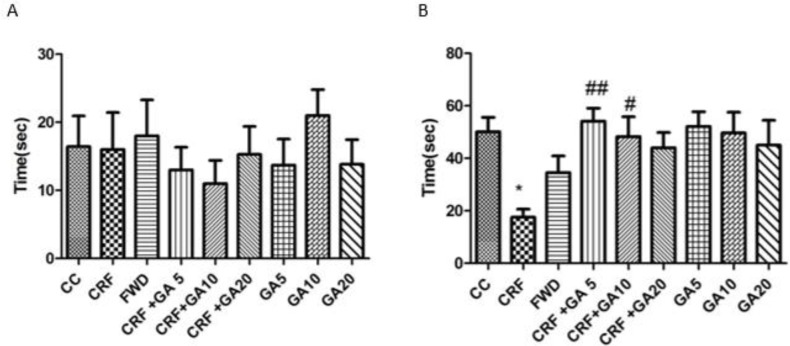
Mean initial (A) and secondary (B) latencies in the shuttle box test; * Shows significant difference compared to control (CC) and # shows significant difference compared to the chronic restraint stress (CRS) group (* *P*<0.05, ** *P*<0.01, and *** *P*<0.001)

**Figure 3.**
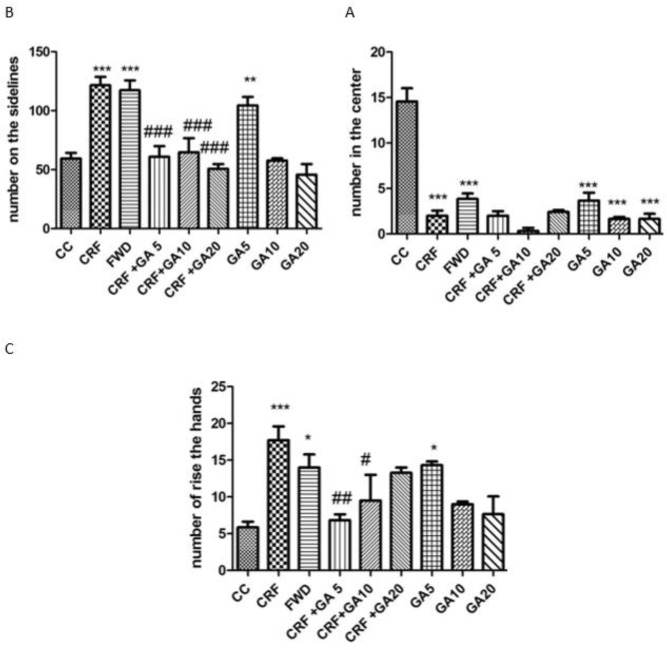
Frequencies of the presence in the central area (A), frequencies of presence in the outer area of the open field (B), and grooming behavior (C); * Shows significant difference compared to control (CC) and # shows significant difference compared to the chronic restraint stress (CRS) group (* *P*<0.05, ** *P*<0.01, and *** *P*<0.001)

**Figure 4:**
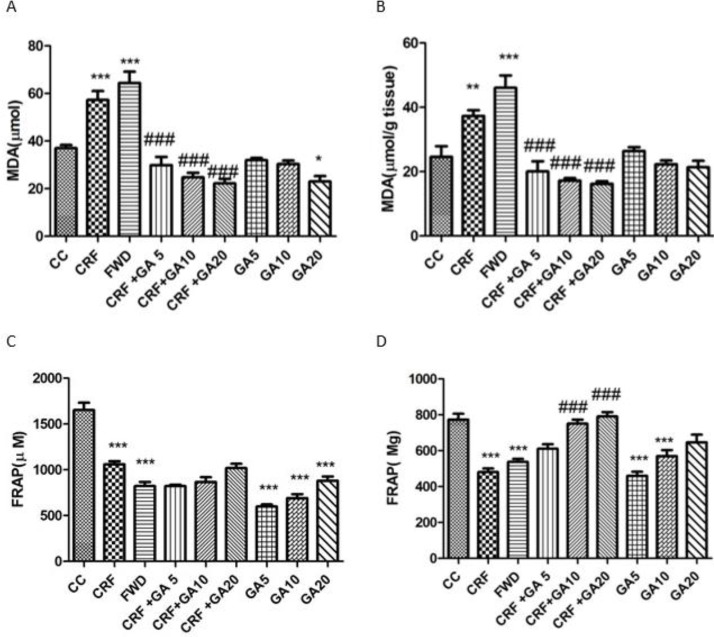
Mean levels of serum MDA (A), brain MDA (B), serum TCA (C), and brain MDA. * Shows significant difference compared to control (CC) and # shows significant difference compared to the chronic restraint stress (CRS) group (* *P*<0.05, ** P<0.01, and *** *P*<0.001)

**Figure 5:**
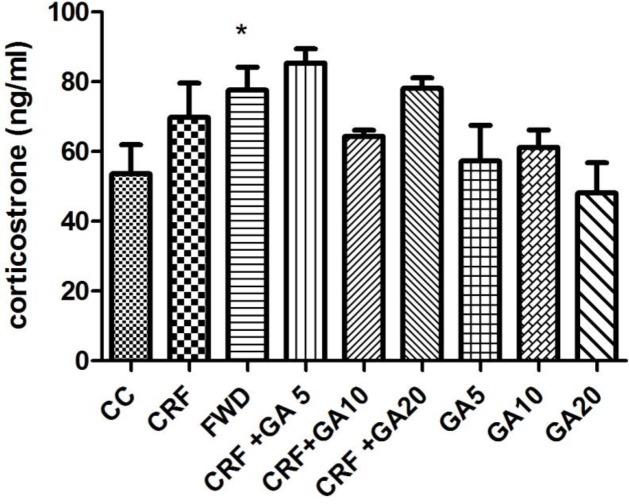
Average serum corticosterone levels in groups. * Shows significant difference compared to control (CC)

*Shuttle box test*

Passive avoidance memory was measured by the shuttle box test. This apparatus has a bright chamber connected to a dark chamber by a guillotine door. Electric shocks are exerted to a conductive metal grid on the floor of the apparatus by a separate stimulus. This test was performed on each mouse for four consecutive days. On the first two days, mice were individually allowed to freely explore the apparatus for 5 min. On the third day, an acquisition test was conducted. Mice were left in the bright chamber and, after 2-min acclimatization, the guillotine door was opened and after the mouse entry into the dark chamber, it was closed and an electrical shock (1 mA/sec) was exerted and the latency to enter the dark chamber was recorded as initial latency. Twenty-four hr later, each mouse was placed in the bright chamber and latency to enter the dark chamber was measured as secondary latency (up to 60 sec) (18).

*Open-field test (OFT)*

OFT is commonly used to study *laboratory animal’s *behavior such as locomotor activity, depression, and anxiety. The apparatus is a quadrant box (30× 30× 15 cm) with the floor divided into squares of 10×10 cm. During the test, a mouse was placed in the center of the floor and its movements were monitored for 6 min. The frequency of mice presence in various regions of the open field was recorded. Grooming behavior was also measured by recording the number of licking or scratching behaviors (17).

*Measurement of the Malondialdehyde (MDA) level*

Two hundred μl of tissue homogenate/ serum was mixed with 1.5 ml of 20% acetic acid, 1.5 ml of 0.8% Thiobarbituric acid, and 200 μl of 8.1% Sodium dodecyl sulfate. The mixture was mixed with 700 µl of distilled water and heated in a boiling water bath for 60 min. After cooling under tap water, distilled water (1 ml) and n-butanol/pyridine solution (5 ml) was added to the reaction mixtures and shaken vigorously. Then, the resulting solutions were centrifuged at 4000 rpm for 10 min and the optical absorbance of the supernatant at 532 nm was recorded (18).

*Measurement of total antioxidant capacity (TCA)*

The total antioxidant capacity of serum and tissue homogenate was measured by using ferric reducing antioxidant power (FRAP) assay. The working FRAP reagent was prepared by mixing acetate buffer (10 ml, 0.25 M, pH= 3.6), 2 TPTZ (5 ml, 10 mM, prepared in 40 mM HCl) and FeCl_3_.6H_2_O (2.5 ml, 20 mM). Twenty five µl of tissue homogenate/serum was added to 1.5 ml of working FRAP solution and left at 37 ^°^C for 10 min. After incubation, the optical absorbance at 593 nm was recorded (18).

*Measurement of serum corticosterone level*

Serum corticosterone level was measured by a corticosterone ELISA Kit (ab108821) according to the manufacturer’s instructions.

***Statistical analysis***

Data were analyzed using SPSS version 20. Analysis of Variance (ANOVA) followed by Duncan’s test was used to identify statistical differences between means. All data were presented as mean±SD and *P*-value less than 0.05 was considered statistically significant.

## Results

Gallic acid showed strong scavenging activity on DPPH radicals (IC_50_=2.15±0.18 µg/ml), strong reducing activity on ferric ion (OD_730__nm_= 1.16-0.30 for 20-100 µg/ml) good scavenging activity on ABTS and hydroxyl radicals (IC_50_= 128.19±21.09 and 601.09±25.06 mg/ml, respectively) and weak chelating activity on ferrous ion (IC_50_>1000 µg/ml).

The number of entries and percentage of time spent in the open and closed arms of EPM are illustrated in Figure 1. As illustrated, CRS and FWD caused a significant reduction in the number of entries and percentage of time spent in the open arms of EPM and a significant increase in the number of entries and percentage of time spent in the closed arms (*P*<0.01 and *P*<0.001). Treatment of CRS mice with gallic acid at a dose of 10 mg/kg significantly reduced the number of open arms entries and reduced the number of closed arms entries (*P*<0.01). Gallic acid at a dose of 20 mg/kg significantly increased the number of open arms entries and reduced the number of closed arms entries (*P*<0.05). Treatment of healthy mice with gallic acid (10 and 20 mg/kg) also induced signs of anxiety.

The mean initial and secondary latencies to enter the dark side of the shuttle box test are shown in Figure 2. There were no statistically significant differences between groups for the initial latency. Exposure to CRS caused a significant decrease in the secondary latency compared to the control group (*P*<0.05). GA at 5 and 10 mg/kg doses caused a significant increase in the secondary latency (*P*<0.05 and *P*<0.01). The secondary latency was not significantly different between healthy control, GA-treated, and FWD groups.

The frequency of grooming behavior and animal presence in the various regions of the open field are illustrated in Figure 3. Mice exposed to 21 days of CRS and FWD showed significant decrease in the frequency of presence in the central area, significant increase in the frequency of presence in the outer area, and significant increase in the grooming behavior (*P*<0.001 and *P*<0.05). Treatment of CRS mice with GA at all three doses caused a significant decrease in the frequency of presence in the outer area (*P*<0.001) but didn’t affect presence in the central area. GA treatment (5 and 10 mg/kg) also caused significant decrease in the frequency of grooming behavior. Treatment of healthy mice with gallic acid also induced anxiety (increased frequency of grooming behavior and presence in the outer region of the open filed and reduced frequency of presence in the central region of the open filed).

MDA and TCA levels in brains and sera of mice are shown in Figure 4. Exposure to CRS and FWD caused significant increase in MDA levels and significant decrease in TCA levels of both serum and brain tissues (*P*<0.001). GA at 10 and 20 mg/kg doses caused a significant increase in brain antioxidant capacity (*P*<0.001). Treatment of CRS mice with all doses of GA significantly reduced serum and brain MDA levels (*P*<0.001).

The serum corticosterone level means are shown in Figure 5. Exposure to CRS and FWD caused increase in blood levels of corticosterone, but the increase was only significant for the FWD group. Treatment with GA did not affect serum corticosterone levels.

**Discussion**

## Discussion

In the current study, we investigated the effect of gallic acid on anxiety-like behaviors and memory impairment induced by CRS. Results indicated that exposure to CRS is associated with memory impairment in shuttle box test and anxiety-related behaviors both in EPM and OFT. These results make sense and are consistent with previous studies (1, 8, 19, 20). In our study, serum corticosterone level showed increase in mice exposed to CRS and FW, but these increases were only significant for WFD mice. CRS and FWD also lead to significant increase in brain and serum lipid peroxidation and significant decrease in brain and serum total antioxidant capacity. 

Increased secretion of glucocorticoids due to the activation of the HPA axis is a prominent and main response to the stress (4). In the brain, the hippocampal structure has the greatest density of mineralocorticoid and glucocorticoid receptors and is highly sensitive to the effects of glucocorticoid hormones in terms of morphology and electrical stimulability (21). Animal studies show that increased blood glucocorticoids during acute and chronic exposure to stress adversely affect memory and learning processes (1, 3, 21). In our study, as expected, CRS and WFD mice had higher serum corticosterone and lower memory function compared to the healthy controls.

Overproduction of reactive oxygen/nitrogen species (ROS/RNS) and related oxidative/nitrosative stress also have been observed in various acute and chronic animal models of stress (22, 23). A high amount of these reactive substances can induce oxidative damage of vital biomolecules such as DNA, proteins, and lipids leading to neural cell death and damage (24). Oxidative damage to neuronal structures associated with learning and memory may impair learning and memory capacity (25). It has also been demonstrated that oxidative stress exposure induces a significant reduction in the number of glucocorticoid receptors in the hippocampal CA1 region (26). Reduced expression of glucocorticoid receptor could impair negative feedback, leading to glucocorticoid hypersecretion and thus further neuronal damage and memory deficits (27).

Concerning the growing evidence on the role of oxidative stress in the development of cognitive and behavioral disorders, it is recommended to use antioxidant compounds as a new approach to treat these conditions (22). In our study, treatment of CRS mice with GA significantly improved passive avoidance memory and reduced anxiety-like behaviors in EPM and OFT tests, GA at a dose of 10 mg/kg showed better efficacy than other doses. It was previously reported that phenolic compounds could ameliorate symptoms of both anxiety and depression by influencing the CNS and decreasing the markers of oxidative damage (28). These compounds also reduce cognitive impairment and boost memory and learning ability (29). Thus, the protective effects of gallic acid on memory impairment of CRS mice may be related to its antioxidant activity. Previous studies also showed the protective effects of GA against oxidative stress induced by streptozotocin (14), transient cerebral ischemia (15), and 6-hydroxydopamine (12). In our study, GA showed promising antioxidant activity both *in vitro* and *in vivo. *It reduced lipid peroxidation and increased antioxidant capacity in sera and brains of CRS mice.

Treatment of healthy mice with GA induced some anxiety-like behaviors in both OFT and EPM tests compared to the control group (caged mice). Also serum and brain antioxidant capacity reduced in GA-treated healthy mice compared to the controls. It seems that GA shows inverse and deteriorative effects in the healthy animals and leads to the development of anxiety and oxidative stress. It has been observed that some herbal extracts with high amounts of polyphenols mediate oxidative stress by interfering with oxidative-antioxidant balance (30).

## Conclusion

GA exerted protective effects against chronic stress-induced mood and cognitive disorders. This can be due to the decrease of lipid peroxidation and increase of TCA in both sera and brains of CRS mice. Treatment of healthy mice with GA had some adverse effects and thus, it is recommended only in the elevated oxidative stress situation.

## Conflicts of Interest

The authors declare that there are no conflicts of interest in this work.
